# Draft genome sequence of the strain 16-537536, isolated from a patient with bronchiectasis and its relationship to the *Pseudomonas koreensis* group of the *Pseudomonas fluorescens* complex

**DOI:** 10.1186/s13104-019-4863-2

**Published:** 2020-01-06

**Authors:** Ad. C. Fluit, Malbert R. C. Rogers, María Díez-Aguilar, Rafael Cantón, Barry J. Benaissa-Trouw, Jumamurat R. Bayjanov, Miquel B. Ekkelenkamp

**Affiliations:** 1 Department of Medical Microbiology, University Medical Center Utrecht, Room G04.614, PO Box 85500, 3508 GA Utrecht, The Netherlands; 2 Servicio de Microbiología, Hospital Universitario Ramón y Cajal, Instituto Ramón y Cajal de Investigación Sanitaria (IRYCIS), Madrid, Spain; 3 Red Española de Investigación en Patología Infecciosa (REIPI), Madrid, Spain

**Keywords:** *Pseudomonas fluorescens*, Bronchiectasis, Whole genome sequencing, Taxonomy, *Pseudomonas koreensis*

## Abstract

**Objective:**

The *Pseudomonas koreensis* group bacteria are usually found in soil and are associated with plants. Currently they are poorly described. Here we report on the whole genome sequence of a bacterial isolate from a patient with bronchiectasis that was first identified as *P. koreensis*, and on its position in the *P. koreensis* group.

**Results:**

Strain 16-537536 was isolated from a patient with bronchiectasis from Spain and initially identified by MALDI-TOF as *P. koreensis*, a member of the *Pseudomonas fluorescens* complex. However, the average nucleotide identity analysis (ANIb) and whole genome alignments of the draft genome sequence of this strain showed it to be a member of the *P. koreensis* group of the *P. fluorescens* complex, but belonging to an undescribed species. In addition, based on ANIb analysis, the *P. koreensis* group contains several other unnamed species. Several genes for putative virulence factors were identified. The only antibiotic resistance gene present in strain 16-537536 was a class C β-lactamase. The correct identification of bacterial species from patients is of utmost importance in order to understand their pathogenesis and to track the potential spread of pathogens between patients. Whole genome sequence data should be included for the description of new species.

## Introduction

The *Pseudomonas koreensis* group is classified within the *Pseudomonas fluorescens* complex and consist of several species, including *Pseudomonas koreensis* [1, 2]. *P. fluorescens* complex isolates have been recovered from many environmental sources, and are frequently associated with plants and soils [3]. *P. koreensis* was first isolated from Korean agricultural soils. Subsequently, it has been isolated from rhizospheres, other agricultural soils [4, 5], and from yak milk [6]. It has also been found as a pathogen in freshwater fish [7]. The bacterium has been reported to produce antimicrobial and antifungal compounds [8–10]. Here we report the sequence of *P. koreensis* group strain 16-537536 from a bronchiectasis patient and on its relationship to other strains of this group for which whole genome sequence data are available.

## Main text

### Methods

Strain 16-537536 was a respiratory isolate from a Spanish patient (50–70 years old) with Karthagener syndrome and bi-basal bronchiectasis in October 2015. It was cultured from a sputum sample by inoculation on 5% sheep blood, chocolate and MacConkey agar plates. Leukocytes and Gram-negative rods were observed in the Gram-stain of the sputum sample. The agar plates were incubated at 37 °C in aerobic and 5% CO_2_ atmosphere conditions; and significant bacterial growth was detected on all the plates after 24 h. Identification and susceptibility testing was performed with the MicroScan Walkaway system (Beckman Coulter), which identified the isolate as *Pseudomonas* spp. The isolate was also analyzed by MALDI-TOF (Bruker, Germany). Briefly, the isolate was spotted in duplo on a steel target and a α-cyano-4-hydroxy-cinnamic acid matrix was applied. The spectra which were obtained were compared to those in the Bruker library. Bacterial DNA was isolated with the QIAcube DNeasy Blood and Tissue Kit using the bacterial or yeast DNA with enzymatic lysis protocol (Qiagen, Germany) after pretreatment with 3 µg/ml lysozyme for 30 min at 37 °C. A DNA library was prepared using the Illumina Nextera XT kit with the corresponding protocol (Illumina, CA) and subsequently sequenced on an Illumina NextSeq platform using the 2 × 150 bp sequencing kit.

Contigs were assembled with SPAdes genome assembler v.3.6.2. Contigs longer than 500 bp with at least tenfold coverage were analyzed further.

The relationship to closely related strains was estimated by establishing an average nucleotide identity score based on BLAST (ANIb) [11, 12], using the whole genome sequences of the *P. koreensis* group strains as previously defined by Garrido-Sanz et al. [13]. In addition, we included the type strain (LMG 21318), two other strains identified as *P. koreensis* (CRS05-R5 and D26) (NCBI GCF_900101414.1, CP015852 and CP014947.1, respectively), and *Pseudomonas baetica* (NCBI PKLC01000000) [14].

The assembled contigs were separately annotated using RAST [15] and Prokka [16]. Further analysis for the presence of resistance genes was performed with ResFinder 3.1 of the Center for Genomic Epidemiology (DTU, Denmark) [17].

## Results

The assembly resulted in 348 contigs with a total length of 6,613,537 bp. The average coverage was 52-fold and the GC-content was 59.9%.

**Fig. 1:**
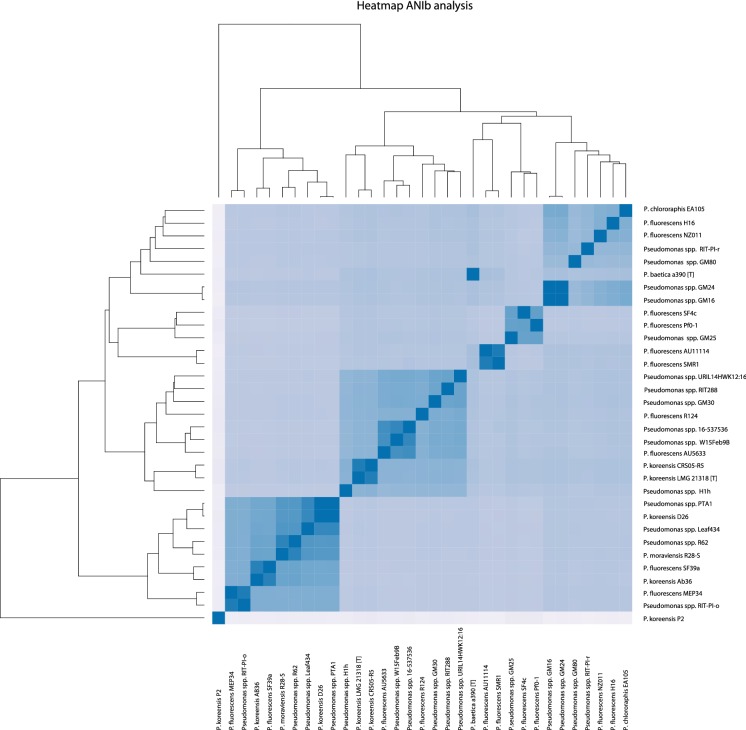
Heatmap of the ANIb of isolates belonging to the *P. koreensis* group of the *P. fluorescens* complex. The identity increases form 85 to 86% (light lavender) to 100% (dark blue). Type strains are indicated by [T] behind the species name. For details of the percentages see Additional file 1: Figure S1

Primary identification was performed by MALDI-TOF. The top 5 results with their score for the first spot were: *P. koreensis* (1.99); *P. koreensis* (1.89); *Pseudomonas jessenii* (1.85); *Pseudomonas azotoformans* (1.82); *Pseudomonas vancouverensis* (1.8) and for the second spot: *P. koreensis* (2.03) *P. jessenii* (1.89); *Pseudomonas corrugate* (1.88); *P. koreensis* (1.84); *P. vancouverensis* (1.82). The single score above 2.00 led to a presumptive identification of the isolate as *P. koreensis*. However, whole genome alignments suggested that *P. koreensis* 16-537536 might belong to a different species. To verify the species identification, an ANIb was performed, using the whole genome sequences of the *P. koreensis* group strains defined by Garrido-Sanz et al. [13]. In addition we included the type strain (LMG 21318) and the sequence data of four other strains identified as *P. koreensis* and available at NCBI (CRS05-R5, P2, Ab36, and D26). When two isolates have an ANIb identity score below 95–96%, they are generally considered to be separate species [11]. Applying a conservative cut-off of 95%, the *P. koreensis group* can be divided into 21 different species (Fig. 1). The ANIb confirms that strain 16-537536 and the *P. koreensis* type strain belong to different, albeit very closely related species. Strain 16-537536 clusters with two strains labeled *P. fluorescens* AU5633 and *Pseudomonas* spp. W15FEB9B. The strain identified as *P. koreensis* CRS05-R5 clusters with the type strain, whereas D26 strain clusters with four other strains, including one that was identified as *Pseudomonas moraviensis*. Strain Ab36 clusters with *P. fluorescens* SF39a. Strain P2 is unrelated to any of the strains tested in the ANIb, with the lowest score when compared to any of the other strains (< 80.3%).

Gene annotation using RAST identified 6194 protein coding sequences and 78 sequences encoding RNAs. An intrinsic class C β-lactamase was identified, but neither RAST nor ResFinder yielded any additional (acquired) antibiotic resistance genes. Based on RAST annotation, there was no evidence for plasmids or transposons. However, alignment against plasmid 4 of *P. koreensis* strain P19E3 (GenBank Acc. No CP027481) showed that 69 kb of DNA encoding putative heavy metal/copper resistance related sequences were also present on the approximately 283 kb P19E3 plasmid. Virulence factors could not be identified using RAST. Annotation with Prokka yielded similar results. Further analysis using BLAST against GenBank identified several putative virulence factors. Although *P. koreensis* group strains are usually not pathogenic in humans, they have been reported as pathogens in freshwater fish [6]. Based on RAST, several potential virulence factors were encoded: enzymes involved in alginate biosynthesis, type II and type VI secretion systems, toxins, pyocins, two filamentous hemagglutinins, urease, fimbriae, a thermostable hemolysin, a flagellum, a microcin, and a bacteriocin.

## Discussion

Strain 16-537536 has a comparable genome size to other *P. koreensis* strains: 6,622,028 bp for CI12, 5,991,224 bp for CRS05-R5, and 6,444,290 bp for the P19E3 chromosome and 1,053,904 bp for its four plasmids ([4, 18]; GenBank Acc. No. CP027477-CP027481).

The results from the ANIb analysis are comparable to those described by Garrido-Sanz et al. [13], with the exception of the strains *Pseudomonas* spp. RIT-PI-o and *P. fluorescens* MEP34, which clustered separately in the study of Garrido-Sanz et al. [13]. The reason for this discrepancy is not clear, but differences between isolate sets may play a role. The ANIb confirms that strain 16-537536 and the *P. koreensis* type strain belong to different, albeit very closely related species.

Our difficulties with identifying strain 16-537536 to the species level, despite having its whole genome sequence available, illustrates the needs for improvement of the the *P. koreensis* group taxonomy. In addition, we found three more strains, currently identified as *P. koreensis*, which potentially represent several novel species. Although the majority of the strains used in our analysis are plant-associated, two isolates were obtained from cystic fibrosis patients and our strain was retrieved from a patient with bronchiectasis; correct species assignment will aid in understanding the epidemiology and pathogenesis of *Pseudomonas* spp. in these diseases. The availability of whole genome sequences of the type strains of all species within the *P. fluorescens* complex would greatly contribute to this objective, as it would allow correct assignment of already sequenced strains and would support the designation of novel species.

The presence of hemolysin has been reported previously for *Pseudomonas aeruginosa* and its activity is considered an important virulence factor in infection [19].

Filamentous hemagglutinin has been shown to be an iron-reponsive virulence factor in the *P. fluorescens* strain TSS, which is pathogenic for fish [20]. A mutant of *P. fluorescens* TSS, in which filamentous hemagglutinin was inactivated, showed less biofilm production and extra-cellular matrix, had no apparent flagella and motility, was defective in attachment to host cells, showed no self-aggregation, exhibited less ability for hemagglutination and had a reduced survival in serum. In vivo experiments in fish showed attenuation of dissemination in tissue by the mutant strain, and reduced host mortality.

Urease has been implicated as a virulence factor during human respiratory tract infection by *Haemophilus influenzae* [21]; the enzyme enhances viability in an acid environment. The urease of strain 16-537536 may play a similar role, especially considering that this strain was isolated from the sputum of a bronchiectasis patient with a lung infection. Based on similarity with proteins in *Pseudomonas* species, strain 16-537536 encodes four potential toxins, one of which was annotated previously as insecticidal toxin complex TcaB2. Additional information for these toxins is lacking and their roles in virulence remains unknown [GenBank accession numbers WP_108183637.1, WP_108183629.1, WP_108183629.1, WP_108181890.1, and WP_108183711.1].

The presence of type II and type VI secretion systems in strain 16-537536 may indicate that the bacterium expresses virulence factors which are excreted by these systems. The type II secretion system of *P. aeruginosa* is a general secretion pathway that secretes virulence factors, such as guanylate cyclase ExoA and the proteases LasA/B; it is also present in other pathogenic bacteria [22, 23]. The type VI secretion systems are used by many Gram-negative bacteria to inject toxic effector molecules into eukaryotic or prokaryotic cells and have a role in eliminating other bacteria that occupy the same niche. This has for example been shown for *Salmonella typhimurium* in an animal model [24]. Pyocins, microcins, and bacteriocins, which are antibacterial peptides, may also contribute to this process [25]. Finally, fimbriae and flagella may contribute to virulence by facilitating adhesion and providing mobility.

Besides these putative virulence factors, additional virulence factors in strain 16-537536 may not been have been identified because annotation of the genome is not complete; many open reading frames were identified as hypothetical proteins. Furthermore, it is unknown whether the identified virulence factors are indeed expressed during human colonization and infection.

## Conclusion

Strain 16-537536 is a member of the *P. koreensis* group and belongs to a novel, currently undescribed species. The strain encodes several putative virulence factors. It has an intrinsic AmpC β-lactamase, but no additional acquired antibiotic resistance genes.

## Limitations

- WGS of type strain not always available.
- Annotation of bacterial genes incomplete.

## Supplementary information

**Additional file 1: Figure S1.** Heatmap of the ANIb of isolates belonging to the *P. koreensis* group of the *P. fluorescens* complex with percentage identity. Type strains are indicated by [T] behind the species name.

## Data Availability

This whole-genome shotgun project has been deposited at ENA under the project number PRJEB30245. The version described in this paper is the first version.

## Abbreviation

ANIb: average nucleotide identity analysis
