# Supplementary material for: Draft genome sequence of the strain 16-537536, isolated from a patient with bronchiectasis and its relationship to the Pseudomonas koreensis group of the Pseudomonas fluorescens complex
Source: BMC Res Notes. 2020 Jan 6;13:10. doi: 10.1186/s13104-019-4863-2 (PMC6945793; doi:10.1186/s13104-019-4863-2)
Supplement: Supplementary file 1 — Additional file 1: Figure S1. Heatmap of the ANIb of isolates belonging to the P. koreensis group of the P. fluorescens complex with percentage identity. Type strains are indicated by [T] behind the species name. [file 13104_2019_4863_MOESM1_ESM.pdf]

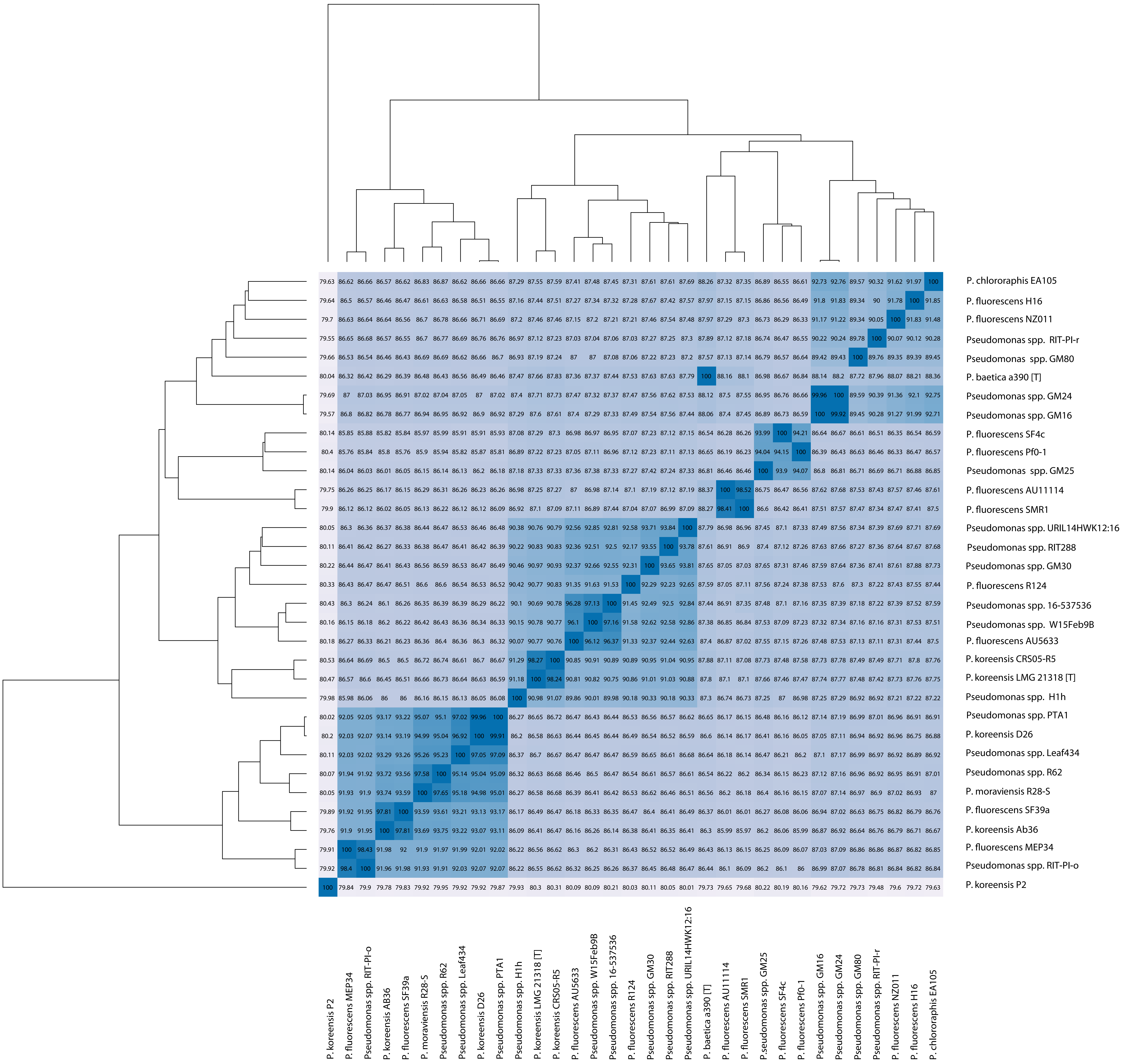

P. chlororaphis EA105
P. fluorescens H16
P. fluorescens NZ011
Pseudomonas spp. RIT-PI-r
Pseudomonas spp. GM80
P. baetica a390 [T]
Pseudomonas spp. GM24
Pseudomonas spp. GM16
P. fluorescens SF4c
P. fluorescens Pf0-1
Pseudomonas spp. GM25
P. fluorescens AU11114
P. fluorescens SMR1
Pseudomonas spp. URIL14HWK12:16
Pseudomonas spp. RIT288
Pseudomonas spp. GM30
P. fluorescens R124
Pseudomonas spp. 16-537536
Pseudomonas spp. W15Feb9B
P. fluorescens AU5633
P. koreensis CRS05-R5
P. koreensis LMG 21318 [T]
Pseudomonas spp. H1h
Pseudomonas spp. PTA1
P. koreensis D26
Pseudomonas spp. Leaf434
Pseudomonas spp. R62
P. moraviensis R28-S
P. fluorescens SF39a
P. koreensis Ab36
P. fluorescens MEP34
Pseudomonas spp. RIT-PI-o
P. koreensis P2
